# Gene expression and fractionation resistance

**DOI:** 10.1186/1471-2164-15-S6-S19

**Published:** 2014-10-17

**Authors:** Eric CH Chen, David Sankoff

**Affiliations:** 1 Department of Biology, University of Ottawa, 30 Marie Curie, K1N 6N5 Ottawa, Canada; 2 Department of Mathematics and Statistics, University of Ottawa, 585 King Edward, K1N 6N5 Ottawa, Canada

**Keywords:** fractionation, whole genome doubling, gene expression, gene function

## Abstract

**Background:**

Previous work on whole genome doubling in plants established the importance of gene functional category in provoking or suppressing duplicate gene loss, or fractionation. Other studies, particularly in *Paramecium *have correlated levels of gene expression with vulnerability or resistance to duplicate loss.

**Results:**

Here we analyze the simultaneous effect of function category and expression in two plant data sets, rosids and asterids.

**Conclusion:**

We demonstrate function category and expression level have independent effects, though expression does not play the dominant role it does in *Paramecium*.

## Background

Whole genome doubling (WGD) is a special case of gene duplication in that everything in the genome, including the genes, regulatory elements, and repetitive regions, is doubled or tripled. This process is more common in plant lineages than in other evolutionary domains [1,2] and is an important source of gene innovations, contributing to diverse morphological and functional complexities in modern plants [3,4]. The duplicated genes are very vulnerable to loss after the WGD event via excision of chromosomal segments or pseudogenization. These losses are collectively referred as fractionation. Various models have been proposed to explain the details of this process, such as the Gene Dosage Hypothesis [5,6] and the Gene Balance Hypothesis [7]. These models try to explain the difference in duplicate gene retention pattern based on the traditional models of gene fate: neofunctionalization, subfunctionalization, and pseudogenization, and on the observations on duplicate gene retentions from WGD.

We have shown in several groups of plants - rosids, asterids, and monocots that the functional category of a gene is a major determinant of fractionation resistance, with metabolic genes being fractionation prone, and "response to stimulus" being fractionation resistant [8,9].

A recurrent theme in works relating to fractionation is the effect of gene expression. In a comprehensive study of fractionation in *Paramecium*, Gout *et al*. [10] identify a clear relationship between high WGD duplicate gene retention rates and high expression level. They also find that within each major gene functional class, higher expression correlates with higher duplicate retention rates; even if the expression levels of each major functional classes differ from each other. They conclude that expression level is the best discriminator for explaining variable resistance to fractionation.

The Gout *et al*. paper [10] is the primary inspiration for this study, where we explore the relationship between of functional class and expression in fractionation resistance in plants. Because we have previously shown that functional class can itself influence the fractionation resistance of the duplicates [8,9] we wish to consolidate these two kinds of findings into one unified framework.

## Results

### Methods

**Figure 1:**
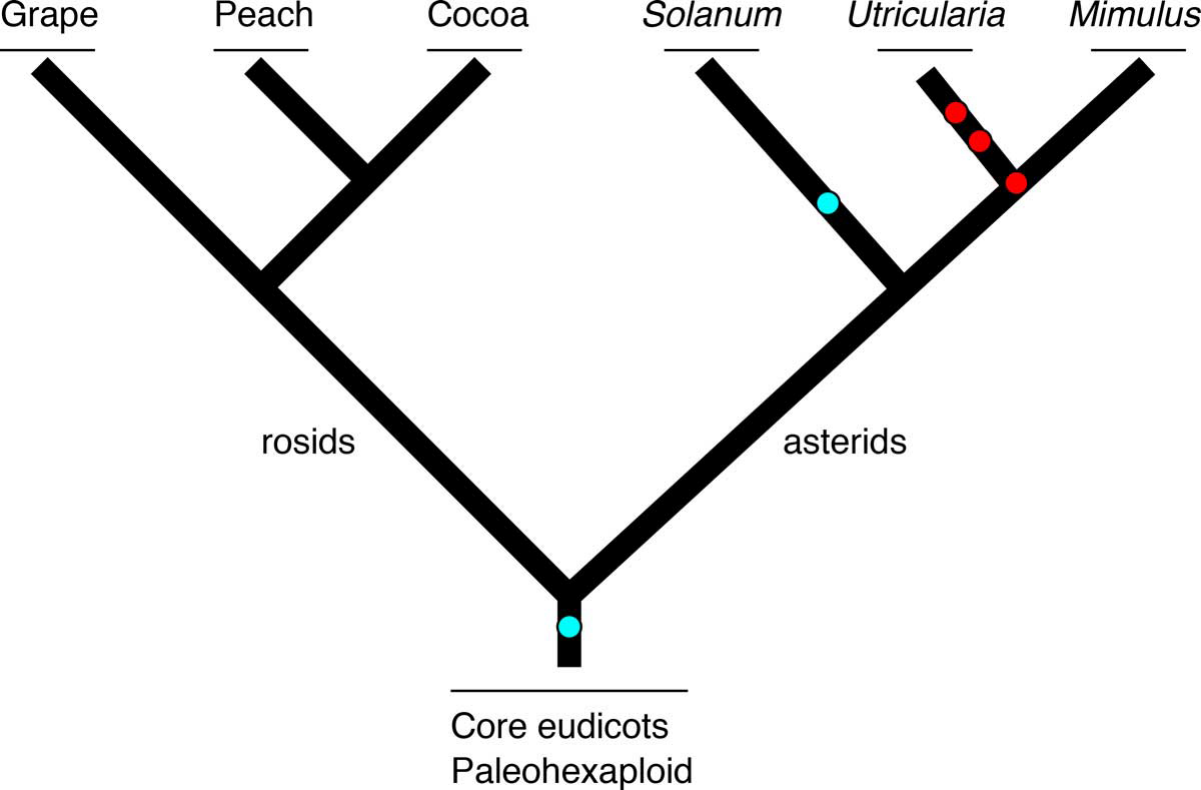
**Phylogeny of the rosids and the asterids data sets**. Species in the rosids data set did not have additional WGD events since their paleohexaploid ancestor. Species in the asterids data set did have additional WGD events (red circles) with one whole genome triplication event (blue circle). Branch length are not to scale.

We analyze the genomes of peach [11], grape [12], and cocoa [13], constituting the rosids data set. These selected species have not undergone WGD events since their triplicated last common ancestor at the base of core eudicots, which makes them invaluable for studying long term effects of fractionation. To study the effect of fractionation from comparatively more recent WGD events and the effect of fractionation from multiple WGD events, we survey the genomes of tomato [14], *Utricularia *[15], and *Mimulus *[16], making up our asterids data set. The asterids diverged from the rosids a few million years after the triplication event in the core eudicots ancestor some 120 million years ago [17] and each of the selected species of the asterids has since undergone more recent WGD events [15] (Figure 1). We will show that even if individual species of each data sets have evolved and fractionated independent of each other, the overall trend of fractionation remains highly parallel.

Due to the still scarce availability of high-quality expression data, we make use of RNA-seq data from grape [18] to represent expression in the rosids and RNA-seq data from tomato [14] to represent expression in the asterids. Tomato gene expression values are about three to four times higher than grape values because of different technology platforms and depth of sequencing. This prevents the meaningful comparisons of absolute gene expression values between grape and tomato. Normalized comparisons, however, are valid.

We are interested in comparing functional categories, expression levels and fractionation among thousands of genes but the inclusion of a few extremely highly expressed genes could swamp some of these comparisons. Thus we filter out the genes in the top 1% of expression levels. The filter is most pertinent to the more specific GO categories such as individual enzyme classes where the number of genes in each class may be small. Filtering is not necessary for the top level categories since they contain thousands of genes, but for consistency we keep the filter for all the expression analysis.

To take into account varying plant tissues having different expression profiles as well as plant responses to different environments and stimuli, we use the highest reported expression value for any given gene rather than the median or the mean. The rationale for this is that the RNA-seq data we are using distinguishes different expression level in different tissues as opposed to responses to a particular stimuli. Many genes are only expressed in specific tissues, so the maximum expression level of a gene is a better indication of its importance in the organism.

### Data

**Figure 2:**
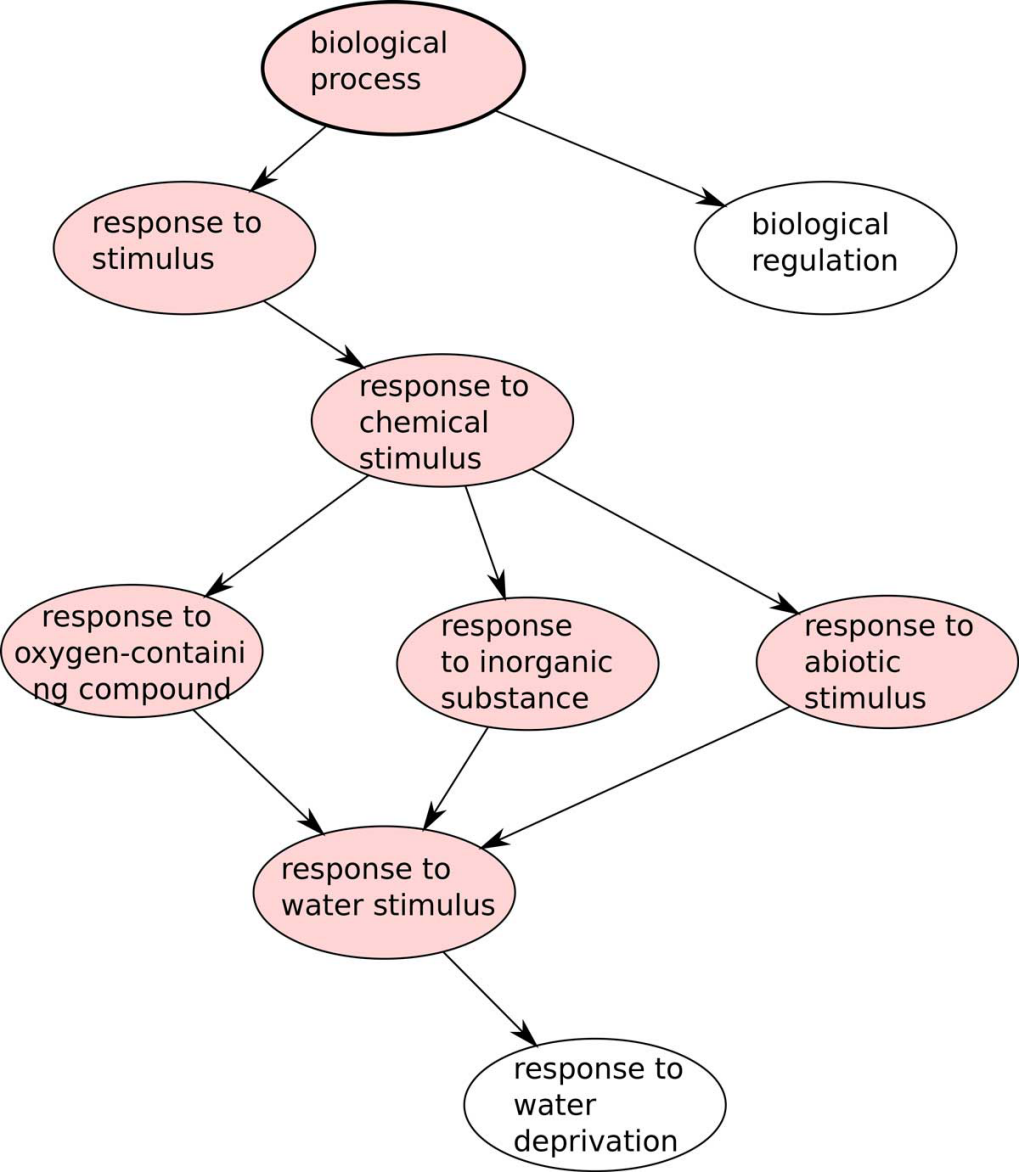
**Gene Ontology**. In this example, if "response to water stimulus" is hit by a gene in a homology set then all the terms above it in the GO hierarchy are also hit (coloured term).

In this comparative study we first categorize genes into distinct homology sets, where each set represents the ancestral gene of the rosids or the asterids ancestor just prior to the WGD event(s) [9,19,20]. Categorization is based on both gene sequence similarities and positional conservation and is done using SynMap [21] with genomic resources obtained from CoGe [22]. The homology sets are refined using algorithms by Zheng *et al*. [19,20]. These homology sets then allow for the calculation of levels of fractionation resistance (*F*). The fractionation resistance of a homology set is determined by the number of species (*N*) that still have the genes of the set in duplicates in the form: *F *= *N *+ 1. The higher the number of species still retaining WGD in duplicates, the higher the fractionation resistance. A gene that has been returned to singleton in all species has *F *= 1. Each homology set is then annotated with Gene Ontology [23] terms using Blast2GO [24] to classify their functional class. All the terms associated with any of the genes in a homology set are retained. In addition, by design of the Gene Ontology [23] if a gene is annotated with a particular GO term, it automatically inherits all the parent GO terms which are more general (Figure 2). Some of our analyses include individual genes rather than whole homology sets; for these we propagate GO annotation by having all genes of a homology set inheriting particular GO terms if one of the genes is annotated with them.

GO terms are divided into three domains "biological process", "cellular component", and "molecular function". A homology set may not always be annotated in all three domains. Most of our analyses will focus on the mutually exclusive top level categories within each domain. Since most of the homology sets have fractionated to be single copies in surveyed species, we use normalized proportions (*P*) to fairly compare enrichment of functional classes. We set *Hit*(*F, C*) = the number of "hits" in category *C *by homology classes within fractionation level *F*.

$$P\left(F,C\right)=Hit\left(F,C\right)/\underset{C^{\prime }}{\sum }Hit\left(F,C^{\prime }\right)$$

Where the sum is taken over all category *C' *in the top level domain including *C*. As we plot *P *(*F, C*) against *F*, we will deem *C *to be fractionation resistant if *P *(*F, C*) increases with increasing *F*. We deem *C *to be fractionation prone if the reverse case is observed, where *P *(*F, C*) deceases with increase in *F*.

A similar formula is used to normalize the distribution of gene expression as well. Log transformed expression level of genes is collected into bins of similar expression levels. Functional expression *E *is a simple average expression of all genes of homology sets of *F *and *C*.

$$P_{B}\left(F\right)=Number\;of\;genes\;in\;bin\;B\;from\;F/\underset{B^{\prime }}{\sum }number\;of\;genes\;in\;F$$

E(F,C)=AverageExpression[GenesofHomologySetin(F,C)]

### Functional analysis

**Figure 3:**
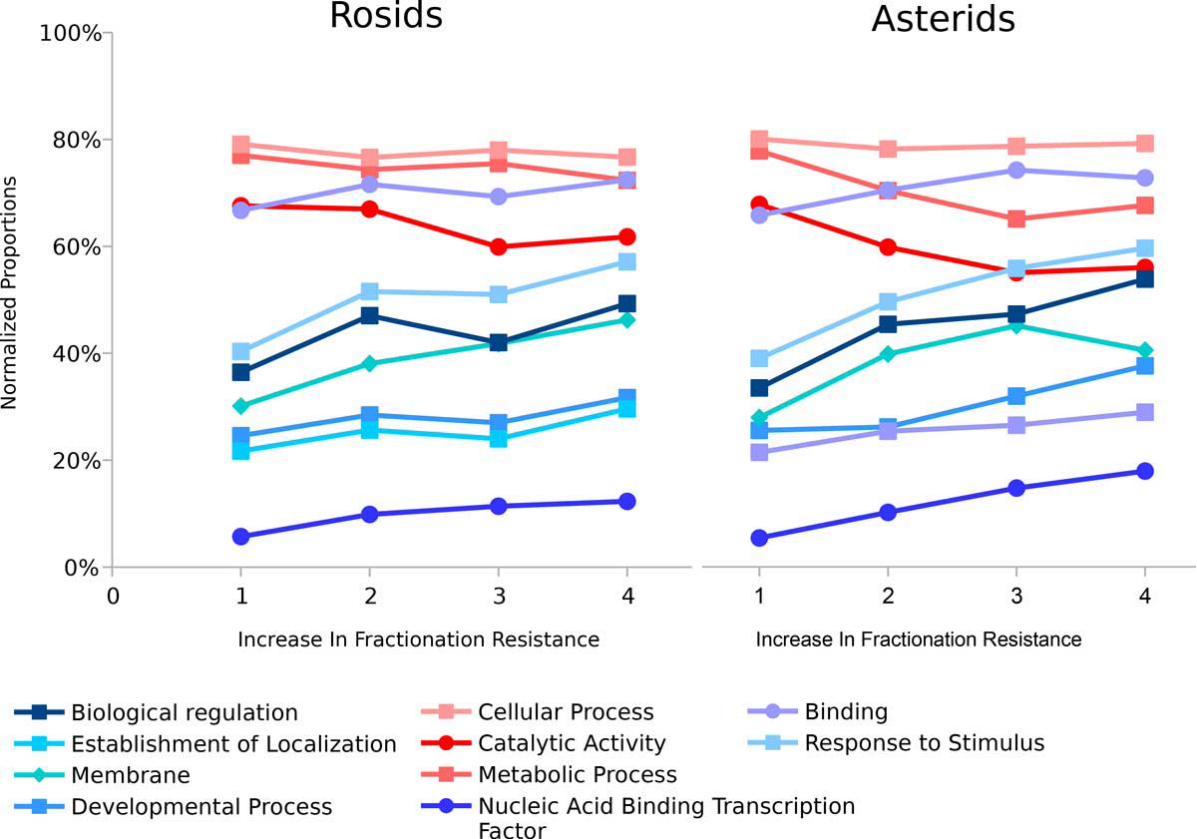
**Biased gene fractionation resistance in different functional class**. Updated result from [9]. Only three functional classes in red are fractionation prone.

Figure 3 displays the effect of functional class on increase in fractionation resistance. The conclusion is that some top level functional classes are more fractionation resistant (such as "biological regulation", "response to stimulus", "membrane", "developmental process", "establishment of localization", and *etc*.) or more fractionation prone (such as "metabolic process", "catalytic activity", and "cellular process") than others and that the finding is highly parallel across these two different lineages. Despite the shallow slopes of the curves on the 0 - 100% scale of normalized proportion, both the rosids and the asterids data sets contain thousands of homology sets and are statistically significant. All functional classes shown have *p *value to be orders of magnitude less than 0.05 in linear regressions. The statistical test is a linear regression test based on all the homology sets, not just the few summary points in Figure 3. This top level result is in agreement with results reported by Gout *et al*. [10] in the organism *Paramecium*.

### Expression analysis

**Figure 4:**
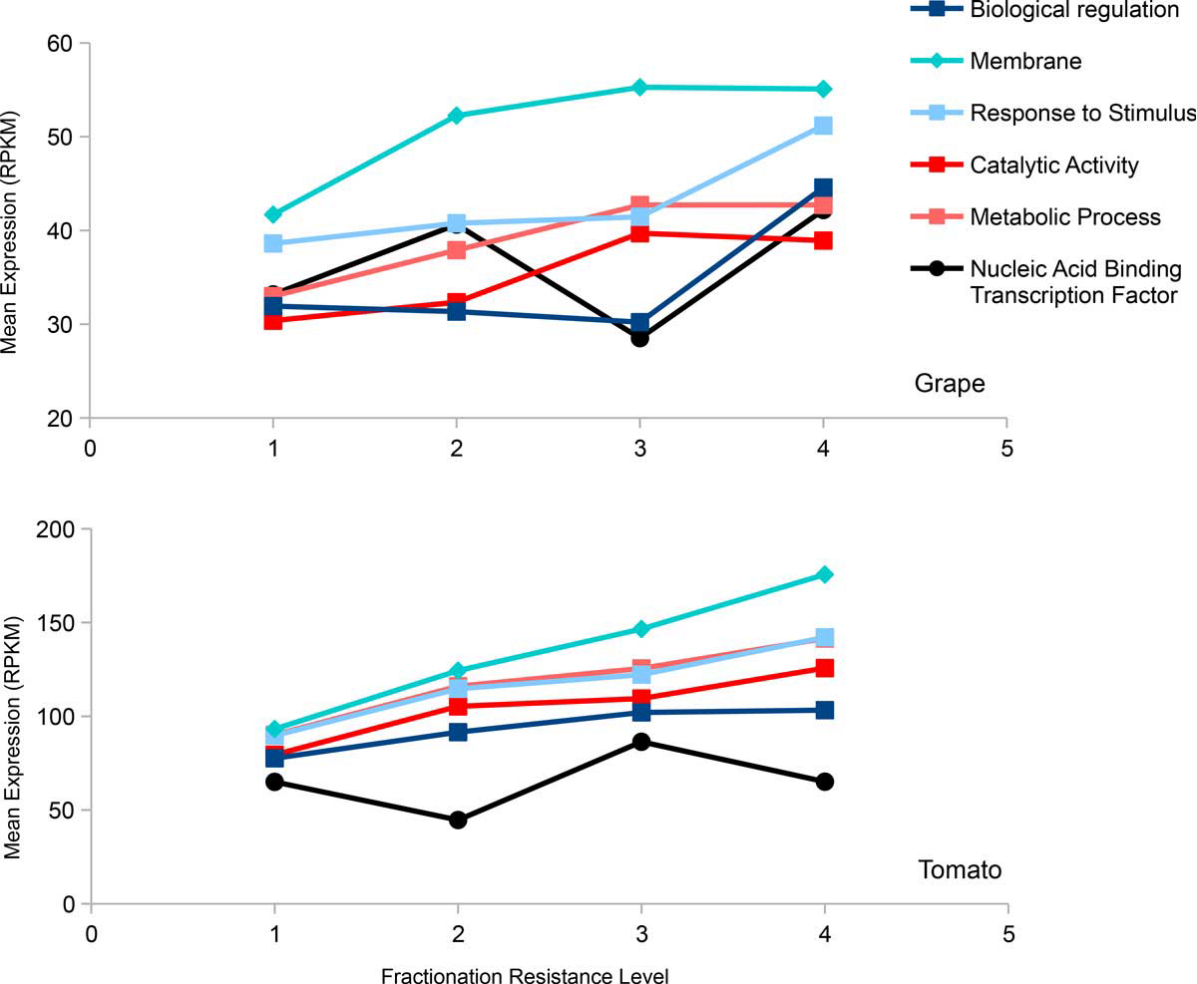
**Gene expression level of different functional class across different fractionation resistance levels**. The units in RNA-seq is expressed as number of reads per kilobase per millions reads (RPKM). "Nucleic acid transcription factor binding activity" (in black) is not significant. The statistical test is based on all genes in a category, not just the few summary points in the graph.

**Figure 5:**
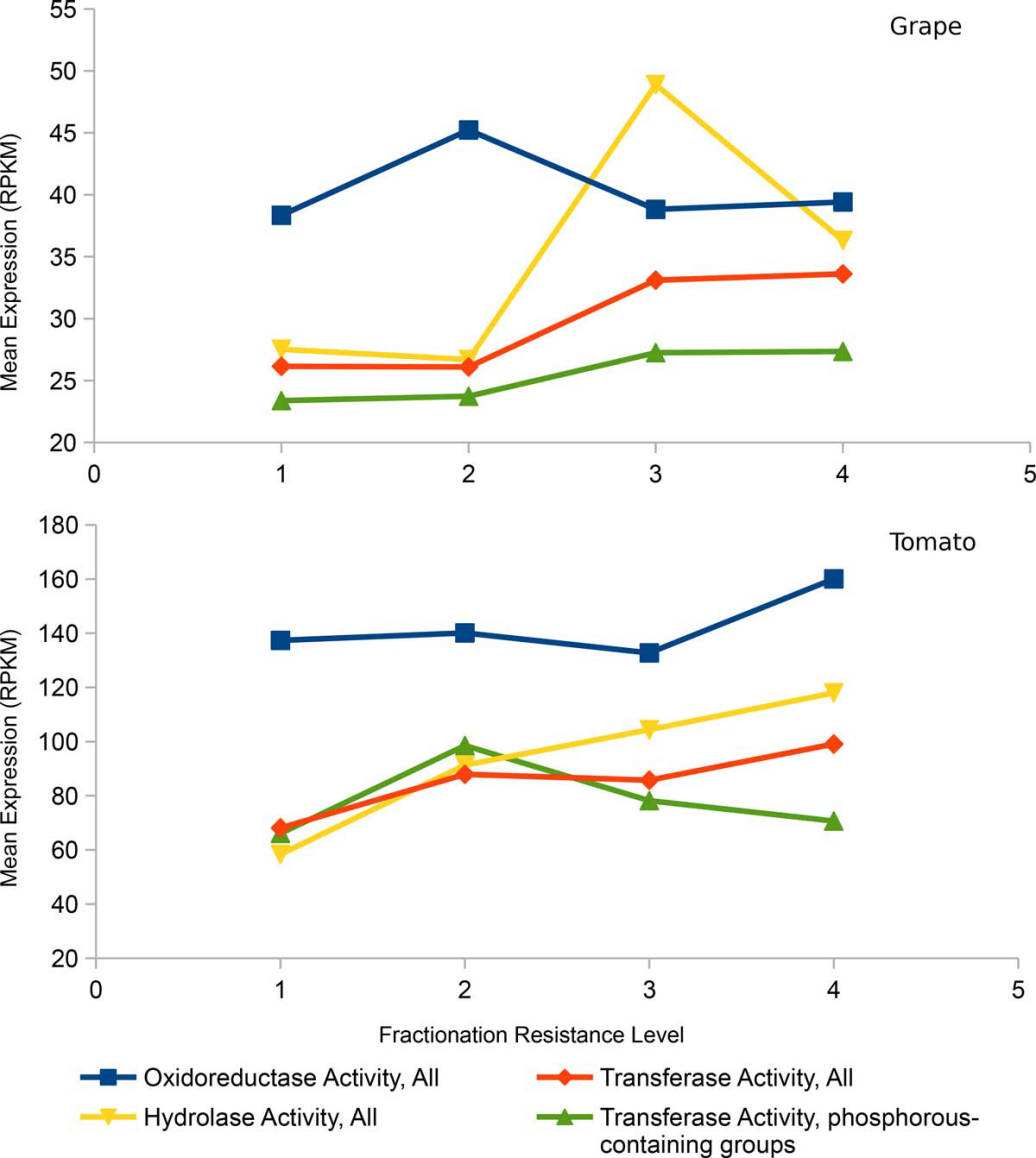
**Expression analysis of major enzyme classes in grape and tomato**. Only showing select enzyme classes that with large member sizes (more than 500 members) that are statistically significant. In enzyme classes with small member sizes, the presence of a few highly expressed genes easily distort the averages, even with the 1% filter in place. The statistical test is based on all genes in a category, not just the few summary points in the graph.

**Figure 6:**
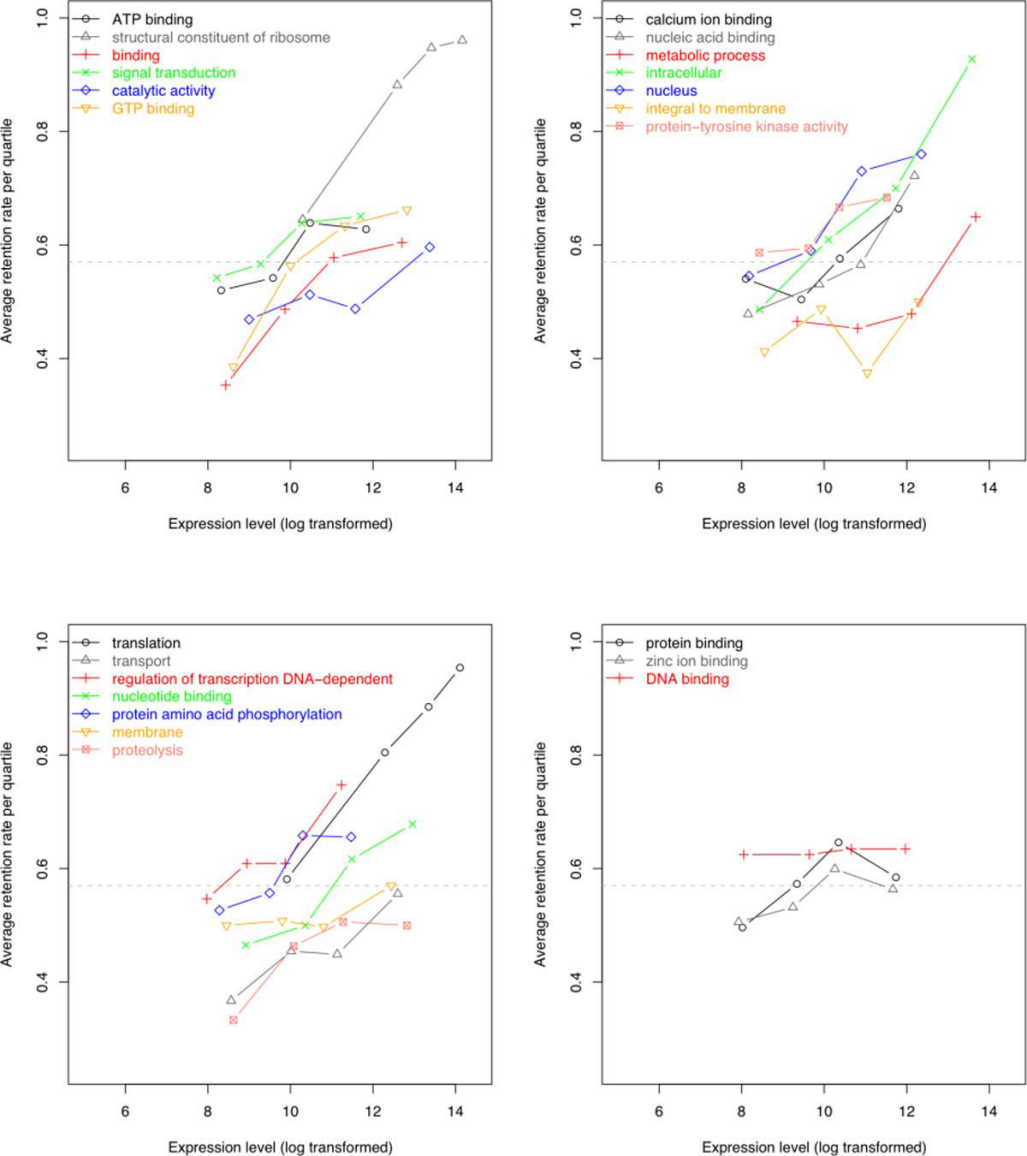
**Taken from Gout et al**. [10] Figure S3

Using means and medians to summarize the expression levels of different fractionation resistant homology sets, which may be represented by one or more genes in grape (or tomato, as the case may be) we also find a trend where more highly expressed sets tend to be more resistant to fractionation. This is true regardless if we plot all genes or plot genes of specific top-level functional class (Figure 4). Investigating more specific functional categories such as enzyme classes (Figure 5) reveal similar findings where higher expression within each class contribute clearly to increase in fractionation resistance. This correlation of higher gene expression to fractionation resistance confirms results reported by Gout *et al*. [10], as reproduced in Figure 6. The startling parallelism between the rosids and the asterids is also present in Figure 4 and 5. The trend of increase in expression level with increase in *F *is the same in both rosids and the asterids. The ranking of functional class by expression to *F *is also the same in both data sets. The effect of expression on *F *appears to be universal.

However, the change in expression levels of sets from the least fractionation resistant to the most fractionation resistant is smaller in our rosids and asterids data sets than in [10]. Functional classes with lower number of genes ("nucleic acid binding transcription factor activity" has 411 genes in grape and 552 genes in tomato) are still suggestive of the trend but are no longer statistically significant in both data sets (*p >*0.05). These differences may be due to sample size rather than the differences between the protist *Paramecium *and plants.

**Figure 7:**
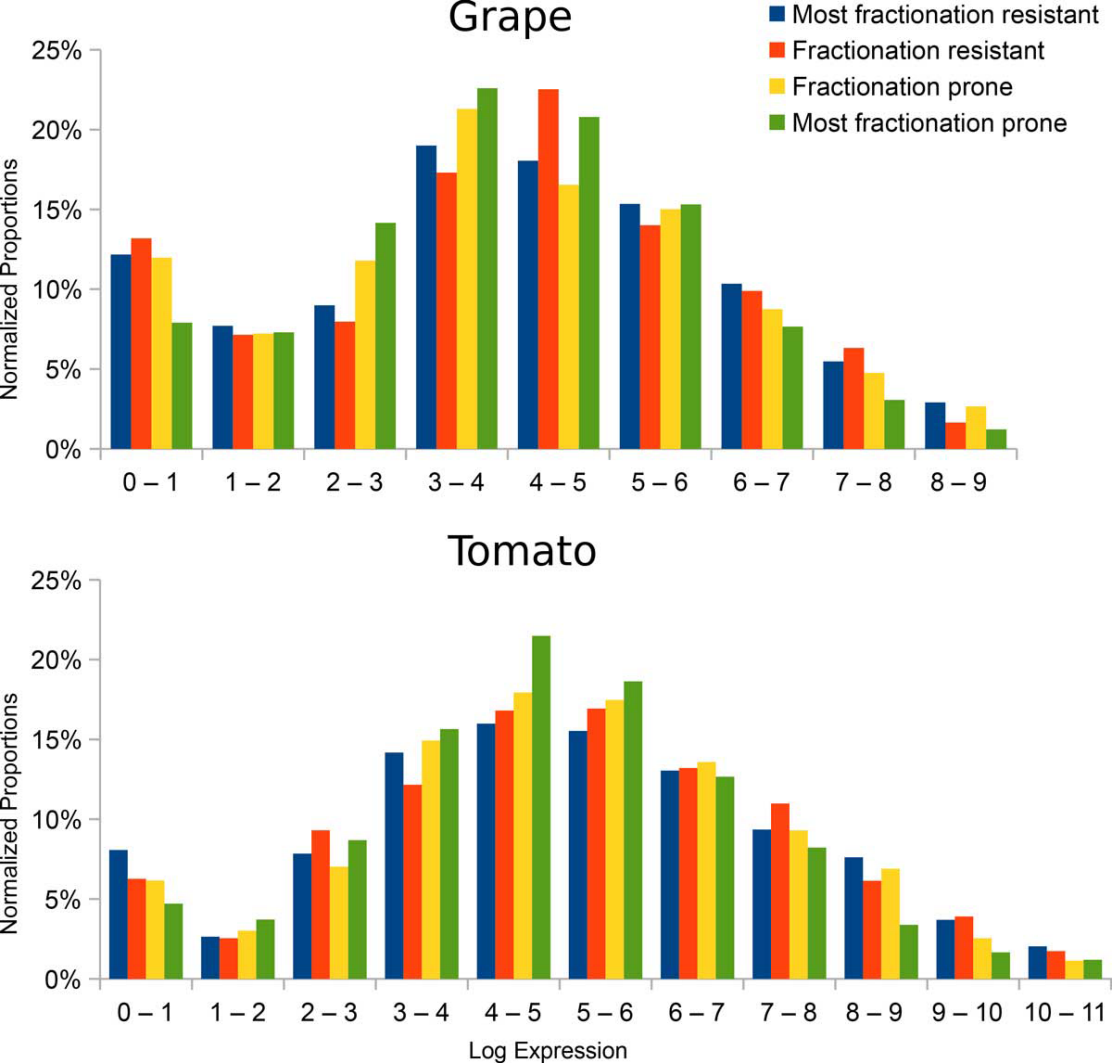
**Gene expression in different fractionation resistance levels**. The nature of a RNA-seq experiment means not all possible states of the organism are recorded, therefore the high proportion in the lowest expression block can be explained by the gene not being activated in the condition when the RNA-seq data was collected.

An important difference between our results and those on *Paramecium *[10] is that a significant number of genes with low expression level are highly retained in the plants whereas in the *Paramecium*, genes with high retention rates are very unlikely to have low expression level. There also exist functional classes that have widely varying expression levels across fractionation resistant class, such as genes from GO term "nucleic acid binding transcription factor activity" (Figure 4). However, using the distribution of gene expression to compare the different fractionation categories instead of using means of categories (Figure 7) reveals that the highest and the lowest expression bins distort the general expression pattern of the rest of genes. Therefore, the data still suggest that higher expression levels result in higher likelihood of being retained in duplicate form.

On the other hand, functional class appears to have greater influence than expression on determining whether a homology set is fractionation resistant or not. Both GO terms "metabolic process" and "catalytic activity", which are reported to be very fractionation prone, have similar expression levels to "response to stimulus", a very fractionation resistant GO term. The GO term "biological regulation", one of the most fractionation resistant terms, has on average lower expression levels than any of the above-mentioned terms in both of the rosids and the asterids datasets.

## Discussion and conclusion

How can we reconcile the relatively small effect of gene expression on fractionation resistance with the claim that gene expression levels are fundamental to copy number variations and fractionation resistance [5,6,10,25,27].

One of the more plausible explanations is rather than just the fitness cost of gene expression controlling fractionation resistance, the fitness cost of disruption of the intended function of the gene or the gene network is a greater contributing factor. Highly connected genes have been reported to be preferentially retained [28,29] and are predicted to be more retained by both the Gene Balance Hypothesis [7] and the Gene Dosage Hypothesis [5,6]. As such, genes in a functional class that generally has low expression levels may still have high fractionation resistance level due to the importance of the function or the functional network.

It should be noted that many other factors have been proposed to explain variable fractionation rates. Moghe *et al*.[30] showed that gene sequence features such as longer amino acid length and higher GC3 level (the wobble position in protein translation), contribute to fractionation resistance in *Raphanus raphanistrum, Arabidopsis thaliana, Arabidopsis lyrata*, and *Brassica rapa *in addition to functional class. They also report that in different WGD events the degree of enrichment from gene sequence features and functional classes may vary, though the directionality of enrichments (be they contributing to fractionation resistance or fractionation proneness) remains mostly the same [30]. We were unable to replicate these results on our data using multiple regression; only expression level was consistently predictive of fractionation.

Of interest, a recent study Makino *et al*. [26] reports the effect of fractionation from ancient vertebrate WGD on the biased distribution of genes with copy-number variations in humans. This paper claims that retained duplicates suppress changes in copy number in their vicinity.

In conclusion our result agrees with current models that expression does play a role in fractionation resistance although by itself it can not explain the enrichments of functional classes. It is likely that systemic analysis on more genomes will be needed to clarify the role of expression and other sequence feature in explaining fractionation. At the present time a good predictor of fractionation resistance should still contain both expression and functional class and may even include how connected a gene is in the genome.

## Competing interests

The authors declare that they have no competing interests.

## Authors' contributions

Both authors participated in the research, wrote the paper, read and approved the manuscript.

## Declarations

The publication charges for this article were funded by the Canada Research Chair in Mathematical Genomics, and by the University of Ottawa.

This article has been published as part of *BMC Genomics *Volume 15 Supplement 6, 2014: Proceedings of the Twelfth Annual Research in Computational Molecular Biology (RECOMB) Satellite Workshop on Comparative Genomics. The full contents of the supplement are available online at http://www.biomedcentral.com/bmcgenomics/supplements/15/S6.
